# 

**Published:** 1835-01-01

**Authors:** 


### THE MEDICO-CHIRURGICAL REVIEW, AND JOURNAL OF PRACTICAL MEDICINE.

*(NEW SERIES.)*

VOLUME TWENTY-TWO,

[1st of OCTOBER, 1834, to 31st of MARCH,]

1835.

VOL. II. of DECENNIAL SERIES.

EDITED

By JAMES JOHNSON, M.D.

PHYSICIAN EXTRAORDINARY TO THE KING,

AND

HENRY JAMES JOHNSON, Esq.

LATE HOUSE SURGEON TO ST. GEORGE'S AND THE LOCK HOSPITALS.

Surgeon Gen'l's Office LIBRARY 57462 Brown

LONDON:

PUBLISHED BY S. HIGHLEY, 32, FLEET STREET,

*Re-printed in New York, by Mr. Wood.*

1835.

W1
MF75T

PRINTED BY G. HAYDEN,
Little College Street, Westminster.

### BIBLIOGRAPHICAL RECORD;

OR,

*Works received for Review since last Quarter.*

1. Ossa Humana. Part IV. Three plates, with numerous Figures. Price 5s. By R. B. CUMMING.

2. Practical Hints on the Treatment of several Diseases. By JOHN PEACOCK, MD. Octavo. pp. 75.

3. The Triumph of Truth & Good Sense; or, an Exposé of Quacks and Quackery. By WILLIAM DE MEY, M.D. &c. Octavo, pp. 30. 1834.

4. Lectures on the ordinary Agents of Life, as applicable to Therapeutics and Hygiene; or the Uses of the Atmosphere, Habitations, Baths, Clothing, Climate, Exercise, Food, Drink, &c. in the Treatment and Prevention of Disease. By ALEX. KILGOUR, M.D. Octavo, pp. 359. Ed. Oct. 1834.

☞ *A good Compilation interspersed with much original reflection and Observation.*

5. A Compendium of Pharmacy, explanatory of the Chemical Decompositions of the Pharmacopœia Londinensis, illustrated by new and comprehensive Diagrams. By WILLIAM MEADE, M.R.C.S. and Private Teacher of Medicine and Surgery. Small 8vo, pp. 147. Jackson, Oct. 1834. Price 4s.

6. On the Anatomy and Diseases of the Neck of the Bladder and of the Urethra; being the Substance of the Lectures delivered in the Theatre of the Royal College of Surgeons in the Year 1830; and in the Westminster Hospital in 1833 and 1834. By G. J. GUTHRIE, F.R.S. Surgeon to the Westminster Hospital, &c. Octavo, pp. 284. Burgess and Hill. Sept. 1834.

---

7. Observations on Functional Affections of the Spinal Cord and Ganglionic System of Nerves, in which their identity with sympathetic, nervous, and simulated Diseases is illustrated. By WILLIAM GRIFFIN, M.D. one of the Physicians to the Limerick County Infirmary and Lying-in Hospital, &c.; and by DANIEL GRIFFIN, M.R.C.S. in London, Surgeon to the Pallas Kenry Dispensary. Octavo, pp. 247. Burgess and Hill. October, 1834.

---

8. The Morbid Anatomy of the Eye. By JAMES WARDROP, Surgeon to the late King. Illustrated by coloured Plates. Two volumes, octavo. Second Edition. Churchill, 1834.

---

9. Dentologia: a Poem on the Diseases of the Teeth, and their proper Remedies. By SOLYMON BROWN, A.M. With Notes, practical, historical, illustrative, and explanatory. By ELEAZAR PARMLY, Dentist. Octavo, pp. 176. New York. 1834.

---

10. A systematic Treatise on Comparative Physiology, introducory to the Physiology of MAN. Translated, with Notes, from the German of FREDERICK TIEDEMANN, Professor of Anatomy and Physiology in the Heidelberg University, by JAMES MANBY GULLY, M.D., and J. HUNTER LANE, M.D. &c. Vol. I. pp. 431. Churchill, London. October, 1834.

☞ *A very valuable Work, which shall be noticed.*

---

11. A Treatise on Diseases of the Chest, and on Mediate Auscultation. From the latest French Edition of M. Laennec, with copious Notes, and a Sketch of the Author's Life. By JOHN FORBES, M.D. &c. Fourth edition, considerably enlarged and improved, with many additional Notes, &c. One volume, octavo, with Plates, pp. 675. Renshaw, 1834.

☞ *This edition is both enlarged and improved. Some of the new notes we shall notice in the Periscope.*

12. An Introduction to the Study and Practice of Medicine: comprising a brief Exposition of the various Branches of Medical Knowledge—Directions for their Study, &c. By JOHN DOWSON, M.D. Octavo, pp. 96. London, 1834.

☞ *There is much judicious advice in this little work, with references to the best authors for the student to consult.*

---

13. An Essay on Clinical Instruction. By A. C. A. LOUIS, M.D. Physician to La Pitié, &c. Translated by PETER MARTIN, M.R.C.S. Octavo, pp. 33. Highley, 1834.

☞ *This little pamphlet is worthy the attention of the pupil entering on attendance at hospitals and dispensaries.*

---

14. The Surgeon's Practical Guide in Dressing, and in the Application of Bandages. Illustrated by numerous Engravings. By THOMAS CUTLER, M.D. late Staff Surgeon in the Belgian Army. Duodecimo, pp, 195, with Plates. Taylor, London, 1834. Price 6s. 6d.

☞ *This is* MULTUM IN PARVO, *and is full of details that cannot be found in any other work.*

---

15. The Elements of Anatomy. By JONES QUAIN, M.D. Professor of Anatomy and Physiology in the University of London. Octavo, pp. 870. Third Edition, revised and enlarged. Taylor, London, Oct. 1834.

---

16. A popular View of Homœopathy. By the Rev. THOMAS EVEREST, Rector of Weckwar, Gloucestershire. Duodecimo, pp. 95. Pickering, London. Oct. 1834.

☞ *We hope the Reverend Divine does not deal out spiritual consolation to his flock, in the homœopathic doses, which he so strenuously recommends physicians to adopt in the administration of medicine to their patients.*

---

17. An Inquiry into the Claims of Dr. William Harvey, to the Discovery of the Circulation of the Blood; with a more equitable Retrospect of that Event; with an Introductory Lecture in Vindication of Hippocrates from the Charge of Ignorance, &c. By JOHN REDMAN COXE, M.D. Professor of Materia Medica in the University of Pennsylvania, &c. Octavo, pp. 258. Philadelphia, July, 1834.

---

18. Introductory Lecture to the Medical Classes of the Charing Cross Medical

School. By William Shearman, M.D. one of the Physicians to the Hospital. Octavo, pp. 16. October 1st, 1834.

☞ *The objects of the institution and the plans of instruction are clearly stated, and some excellent advice to students is interwoven with other matters.*

19. A Manual for Students who are preparing for Examination at Apothecaries' Hall. By John Steggall, M.D. Sixth Edition. Small 8vo, pp. 302. Churchill, London, Oct. 1834.

☞ *A sixth edition requires no comment. Some important notes have been added to the present edition, as well as alterations and improvements.*

20. A Treatise on Dropsy, exhibiting its Nature, Causes, Forms, Symptoms, Principles of Treatment, &c. By James Ford, M.D. Octavo, pp. 57. Oct. 1834.

21. A Manual of Aphorisms in Chemistry; the Chemico-pharmaceutical Preparations and Decompositions of the London Pharmacopœia, and Toxicology, &c. By Robert Venables, M.D. &c. Small 8vo, pp. 251, price 7s. Highley, Fleet-st. 1834.

☞ *A capital manual for Apothecaries, Hall.*

22. Principles and Practice of Obstetric Medicine, &c. Parts XXXVI. & XXXVII. By D. D. Davis, M.D.

☞ *The diseases of the female breast are continued in the 36th Fasciculus, and ably treated of. The 37th part takes up the subject of pregnancy.*

23. An Inquiry into the Nature and Properties of the Blood, in Health and in Disease. By the late Charles Turner Thackrah. A new and enlarged Edition, arranged and revised by Thos. G. Wright, M.D. To which is prefixed, a Biographical Memoir of Mr. Thackrah. Octavo, pp. 247. November, 1834.

24. The Cyclopædia of Practical Medicine. Part XXIII. Containing Tubercular Phthisis, by Dr. Clarke—Tympanitis, by Dr. Kerr—Morbid States of Urine, by Dr. Bostock—Bloody Urine, by Dr. Goldie—Urticaria, by Dr. Houghton—Pathology of the Uterus, by Dr. Lee—Vaccination, by Dr. Gregory—Diseased Valves of the Heart, by Dr. Hope—Diseases of Veins, by Dr. Lee—Ventilation, by Dr. Brown.

☞ *There are some capital articles in this part.*

25. The Principles of Ophthalmic Surgery; being an introduction to a knowledge of the Structure, Functions, and Diseases of the Eye, &c. By John Walker, Assistant Surgeon to the Manchester Eye Institution. Small 8vo, pp. 195, price 5s. 6d. Taylor, London, November, 1834.

☞ *This little work presents a very condensed view of the chief points of treatment of ophthalmic complaints. It is a very useful vade-mecum for students attending the ophthalmic institutions.*

26. The Gums; with late Discoveries on their Structure, Growth, Connexions, Diseases, and Sympathies. By Mr. George Waite, Member of the Royal College of Surgeons, London. Small 8vo, pp. 160. December, 1834.

27. A Series of Anatomical Plates, &c. By Jones Quain, M.D. Division I. Muscles. Fasciculus XIX. Dec. 1834.

28. The Medical Pocket-book for 1835, containing a Case-book and Almanack, with an Account of Medical Corporations, &c. By John Foote, Jun. Renshaw, Dec. 1834.

☞ *This pocket-book contains a great deal of useful information for the medical practitioner.*

29. Dr. Quain's XX. Fasciculus of Anatomical Plates is just received. As also the 38th part of Dr. Davis's Principles and Practice of Obstetric Medicine.

*(In the press.)* A Translation of M. Louis's Researches on Phthisis, with Notes, Additions, and an Introduction. By Chas. Cowan, M.D.

*(In the press.)* Observations on the Causes and Treatment of Ulcerous Diseases of the Leg. By Mr. J. Spencer.

*(In the press.)* Observations on the Medical Charities of Ireland, with a Plan of a Medical Poor-law for the support of a National System of Infirmaries, Fever-hospitals, and Dispensaries, by which the really Sick-poor in every Parish in the Kingdom can have efficient Medical Aid." By Denis Phelan, Esq. of Clonmell. To be published in February next.

